# Humanistic burden and medical care patterns of real-world patients with myasthenia gravis in Japan

**DOI:** 10.3389/fneur.2025.1673297

**Published:** 2025-12-11

**Authors:** Mami Kasahara-Kiritani, Yosuke Saga, Sayuri Watanabe, Thomas Webb, Keira Herr, DaeYoung Yu, Ciara Ringland, Shiva Lauretta Birija, Joe Conyers, Gregor Gibson, Niall Hatchell, Nan Li

**Affiliations:** 1 Integrated Market Access Division, Johnson & Johnson, Tokyo, Japan; 2 Medical Affairs Division, Johnson & Johnson, Tokyo, Japan; 3 R&D Global Development, Johnson & Johnson, Tokyo, Japan; 4 Johnson & Johnson, Singapore, Singapore; 5 Johnson & Johnson, Seoul, Republic of Korea; 6 Adelphi Real World, Bollington, United Kingdom

**Keywords:** generalized myasthenia gravis, humanistic burden, treatment patterns, cross-sectional survey, health-related quality of life

## Abstract

**Introduction:**

Myasthenia gravis (MG) is a rare autoimmune disease characterized by skeletal muscle weakness. As limited real-world data are available in Japan, we aimed to describe the humanistic burden of disease (primary aim), mainly with regards to health-related quality of life (HRQoL), and treatment patterns (secondary aim) in patients with generalized MG (gMG).

**Methods:**

Data were drawn from the Adelphi Real World MG Disease Specific Programme™, a cross-sectional survey of neurologists and their patients in Japan from August 2023 and January 2024. Analyses were descriptive.

**Results:**

Overall, 40 neurologists reported data for 128 patients, where 29 patients had self-reported data. Mean (standard deviation) patient age was 57.9 (16.0) years and 53.9% were female. At data collection, 98.4% of patients were receiving maintenance therapy (including novel treatments). Nonsteroidal immunosuppressant therapies were used at first-line of therapy in 54.5% of cases (*n* = 67/123). Oral systemic steroids were most used [78.0% of patients at first-line (*n* = 96/123), 77.9% at second-line (*n* = 53/68), and 75.0% at third-line (*n* = 15/20)]. The median (IQR) duration from symptom onset to diagnosis was 2.0 (0.9–4.3) months. Of 28 patients with EQ-5D-5L data, 46.4% reported difficulties with usual activities, 42.9% with mobility, 21.4% with self-care, 53.6% with pain/discomfort, and 39.3% with anxiety/depression.

**Conclusion:**

Most patients in this Japanese cohort with gMG received maintenance therapy and the time from symptom onset to diagnosis was relatively short. However, impaired HRQoL remained.

## 1 Introduction

Myasthenia gravis (MG) is a rare autoimmune disease characterized by skeletal muscle weakness caused mainly by antibodies against the acetylcholine receptor (AChR), and less commonly by antibodies against muscle-specific kinase (MuSK) or other AChR-related proteins, at the neuromuscular junction membrane (1–4). Typically, most patients who initially present with ocular symptoms (e.g., ptosis and/or diplopia) develop generalized disease over time, with fluctuating fatigable weakness of the limb, bulbar and respiratory muscles (2, 3, 5, 6). MG has been shown to have a considerable negative impact on health-related quality of life (HRQoL), including physical and social functioning, and mental and emotional health (2, 5, 6).

An interim analysis of an ongoing international, digital, observational study revealed patient-reported problems with usual activities, anxiety and depression, tiredness, breathing and vision, with the impact of MG increasing substantially with increasing disease severity (5). More recent data from the same study showed that despite current treatments, patients still experienced moderate burden, with considerable impact of emotional/psychological comorbidities (2). In Japan, a recent qualitative survey of MG patients' perspectives identified several unmet needs in this population, with patients reporting that symptoms had a significant impact on their daily lives, including their ability to exercise, work, and socialize with friends. Overall, 27% of patients surveyed were dissatisfied with life (6). Similar findings had been reported in a previous patient survey in Japan, which revealed social disadvantages in a substantial proportion of patients (27% and 36% had experienced unemployment and a decrease in income, respectively), with half of patients also reporting feeling reduced social positivity (7).

The estimate of MG patients in Japan is 30,000 (1, 8). The current Japanese clinical guidelines (1) recommend early fast-acting treatment (EFT) for generalized myasthenia gravis (gMG), which is effective for early achievement of minimal manifestations (MM) with oral prednisolone of ≤ 5 mg/day (MM-5 mg), the main goal of treatment. However, a recent retrospective, observational database study of gMG patients in Japan revealed that the target daily dose of 5 mg/day was exceeded by 70% of oral steroid-treated patients (26% exceeded 10 mg/day) (9), highlighting a continued reliance on oral steroids for the management of gMG in Japan. Fast-acting treatment (FT) incorporates plasmapheresis, intravenous immunoglobulins (IVIg), intravenous methylprednisolone (IVMP), or a combination of these. Cases which require frequent FT (e.g., three or more times per year) are considered refractory. With current treatment options—mostly steroids and immunosuppressive agents (10, 11)—around 10%−20% of patients fail to respond adequately (10–12).

Given the limited published real-world evidence in Japan, especially beyond patient-reported data, the primary objective of this study was to describe the humanistic burden of disease with regards to physical symptoms and HRQoL, in a cohort of patients with gMG in Japan. A secondary aim was to describe treatment patterns, diagnostic pathway, and healthcare resource utilization in this population.

## 2 Methods

Data were sourced from the Adelphi Real World MG Disease Specific Programme (DSP)™, a cross-sectional survey with retrospective data capture of physicians (neurologists), patients with gMG and their caregivers conducted in Japan between August 2023 and January 2024.

The DSP methodology has been previously described (13, 14), validated (15), and demonstrated to be representative and consistent over time (16).

### 2.1 Participants

Neurologists were identified by local fieldwork agents using physician panels and publicly available lists and invited to participate if they were actively involved in the management of patients with gMG and were seeing a minimum of one patient with gMG per month.

Each physician completed a detailed electronic patient record form (PRF) for up to five consecutively consulting patients with gMG, capturing patient demographics, clinical characteristics, diagnostic testing and treatment patterns.

These patients were invited to voluntarily complete a “pen and paper” patient self-completion (PSC) form assessing HRQoL and satisfaction with treatment. Patients were eligible for inclusion if they were aged ≥18 years, had a physician-confirmed diagnosis of gMG, and were not participating in a clinical trial at data collection.

If a caregiver was present at the patient's consultation with their physician, they were invited to complete a “pen and paper” caregiver self-completion (CSC) form assessing caregiving requirements and HRQoL. Caregiver inclusion required the caregiver to be ≥18 years at data collection and to have attended the consultation with the patient for which a PRF was completed.

### 2.2 Assessment tools

The following assessment tools were used to evaluate the severity of disease and the humanistic burden of gMG on patients (physician- and/or patient-reported) or caregivers (caregiver-reported), as appropriate.

The Myasthenia Gravis Foundation of America (MGFA) clinical classification system was used to determine patients' disease severity, from Class I (patients with ocular MG alone) to Class V (patients with a myasthenic crisis) (5, 17). For analysis, Class IIa and Class IIb were combined, as well as Class IIIa and Class IIIb to provide a larger sample. In this study, the MGFA was determined by each patient's neurologist.

The Myasthenia Gravis Composite (MGC) score (18) includes 10 test items (a mixture of physician-reported and patient-reported test items) that measure symptoms and signs of MG, with weighted response options (19). The total score ranges from 0 to 50, with higher scores indicating more severe impairments.

The Myasthenia gravis activities of daily living scale (MG-ADL) (20) is an eight-item patient-reported MG-specific questionnaire assessing symptoms across four domains (bulbar, respiratory, limb weakness, and ocular). Scores range from 0 (no impact) to 24 (severe impact) (5).

The myasthenia gravis-quality of life 15-revised (MG-QOL-15R) (21) is a MG-specific 15-item patient-reported HRQoL questionnaire assessing the impact of MG across four domains: emotions, physical health, self-care, and social life and role. The score ranges from 0 to 30, with higher scores indicating greater impact on HRQoL (5).

The EQ-5D-5L (22) is a generic, standardized patient-administered HRQoL instrument comprising five dimensions of health (mobility, self-care, usual activities, pain/discomfort, and anxiety/depression). Scores are given as a single index “utility” value ranging from < 0 (health state considered to be worse than death), to dead (0) and full health (1) (5). The current study used the Japanese value set described by Shiroiwa et al. (23). The EQ-5D visual analog scale (VAS) was used to record the patient's self-rated health, from 0 (worst imaginable health state) to 100 (best imaginable health state) (22).

The functional assessment of chronic illness therapy—fatigue scale (FACIT-fatigue) (24, 25) is a 13-item questionnaire assessing self-reported fatigue and its impact on daily activities and function. Each item is measured on a four-point Likert scale and the total score ranges from 0 to 52, with higher scores representing less fatigue.

The Zarit burden interview—short form (ZBI-12) (26, 27) is a 12-item self-administered questionnaire, which is used to evaluate the impact of disease on caregivers. The ZBI assesses the caregivers' overall QoL, emotional wellbeing, and impact on social and family relationships. Each item is rated on a 5-point Likert scale and the score ranges from 0 to 48. A total score >17 indicates a high burden.

### 2.3 Definitions

In the present study, the following definitions were used to determine remission status at data collection:

- Complete stable remission: the patient has had no symptoms or signs of MG for at least 1 year and has received no therapy for MG during that time. There is no weakness of any muscle on careful examination by someone skilled in the evaluation of neuromuscular disease (isolated weakness of eyelid closure is accepted).
- Pharmacological remission: the same criteria as for complete stable remission except that the patient continues to take some form of therapy for MG. Patients taking cholinesterase inhibitors were excluded from this category because their use suggests the presence of weakness.
- MM-5 mg: minimal manifestations with oral prednisolone of ≤ 5 mg/day.
- No substantial decrease in clinical manifestations.

Regarding the definition of line of MG treatment in this study, the PRF specified the following: “Please consider a new regimen as the start, stop or switch of any treatment, please do not include treatment breaks.”

### 2.4 Statistical analysis

All data were de-identified and anonymized before they were received by Adelphi Real World. Data were analyzed using UNICOM^®^ Intelligence Reporter version 7.5 (28).

Results are presented as frequency (%), mean and standard deviation (SD), and/or median and interquartile range (IQR).

All completed PSCs were matched to a PRF provided by a physician. All completed CSCs were also matched to a physician-provided PRF.

Missing data were not imputed and therefore the base could vary between variables—this is reported separately where appropriate.

## 3 Results

### 3.1 Physician characteristics

Forty neurologists completed PRFs for 128 patients with gMG in Japan.

Physicians reported seeing a mean (SD) of 14.0 (16.4) patients with gMG in a typical month. They also indicated that a mean (SD) of 71.2% (41.3%) of their patients were seen in a community hospital, 26.8% (41.1%) in an academic hospital, 1.4% (7.9%) in an office setting, and 0.6% (4.0%) in another setting (classified as “other”).

Most physicians (70.0%) stated that they had never been involved in a clinical trial for MG.

### 3.2 Patient demographics and characteristics

Of 128 patients included in the study, 29 patients (22.7%) also had self-reported data. Twelve caregivers completed CSCs.

Patients' demographics and clinical characteristics are summarized in Table 1.

**Table 1:** Physician-reported patient demographics and characteristics.

**Variable**	**At time of data collection**
**Age (in years)**	*n* = 128
Mean (SD)	57.9 (16.0)
**Sex**, ***n*** **(%)**	*n* = 128
Male	59 (46.1)
Female	69 (53.9)
**Body weight (in kg)**	*n* = 128
Mean (SD)	58.5 (13.1)
**Time since diagnosis, months**	*n* = 114
Median (IQR)	65.6 (26.8–105.6)
**MGFA classification**, ***n*** **(%)**	*n* = 128
Class I	36 (28.1)
Class II	76 (59.4)
Class III	10 (7.8)
Class IV	5 (3.9)
Class V	1 (0.8)
**MG autoantibody status**, ***n*** **(%)**	*n* = 128
Anti-AChR positive	115 (89.8)
Anti-MuSK positive	5 (3.9)
Anti-LRP4 positive	2 (1.6)
Anti-Kv1.4 positive	1 (0.8)
Another autoantibody (specify)	1 (0.8)
Patient is seronegative (no detectable antibody)	5 (3.9)
**MG-ADL total score**	*n* = 128
Mean (SD)	4.2 (4.0)
**MGC total score**	*n* = 128
Mean (SD)	6.8 (8.4)
**Comorbid conditions**, ***n*** **(%)**	*n* = 128
Yes	77 (60.2)
No	51 (39.8)
**Most common comorbid conditions**, ***n*** **(%)**	*n* = 77
Hypertension	28 (36.4)
Dyslipidemia	22 (28.6)
Diabetes without chronic complications	13 (16.9)
**Employment status**, ***n*** **(%)**	*n* = 120
Working full-time	42 (35.0)
Working part-time	13 (10.8)
On sick leave	4 (3.3)
Homemaker	19 (15.8)
Student	1 (0.8)
Not working due to retirement	15 (12.5)
Unemployed	26 (21.7)

AChR, acetylcholine receptor; IQR, interquartile range; Kv1.4, voltage-gated potassium channel subfamily A member 4; LRP4, lipoprotein receptor-related protein 4; MG, myasthenia gravis; MG-ADL, myasthenia gravis activities of daily living scale; MGFA, myasthenia gravis foundation of America; MGC, myasthenia gravis composite; MuSK, muscle-specific kinase; PRF, patient record form; SD, standard deviation.

The mean (SD) patient age was 57.9 (16.0) years, 53.9% of patients (*n* = 69) were female, and 60.2% of patients (*n* = 77) had not been diagnosed with any comorbid conditions. Mean (SD) body weight was 58.5 kg (13.1). The most common comorbid conditions were hypertension (36.4% of patients) and dyslipidemia (28.6%). Almost half of patients were working full- or part-time [*n* = 55/120 (45.8%)], while 37.5% (*n* = 45/120) were either unemployed, retired, or on sick leave.

The median (IQR) time since gMG diagnosis was 65.6 (26.8–105.6) months. A total of 59.4% of patients (*n* = 76) were classified as MGFA Class II at data collection, and 89.8% of patients (*n* = 115) were autoantibody-positive for AChR. Mean (SD) MG-ADL and MGC total scores were 4.2 (4.0) and 6.8 (8.4), respectively. Supplemental Table 1 shows these scores by MGFA classification.

The most reported symptom at data collection was ptosis (73.4% of patients), followed by diplopia (56.2%; Table 2). These were also considered the most bothersome symptoms [ptosis: 81.7% of patients (*n* = 94/115); diplopia: 62.6% (*n* = 72/115); Table 2].

**Table 2:** Physician-reported most common and most bothersome symptoms.

**Variable**	**At time of data collection**
**Most common symptoms (reported**	*n* = 128
**in** >**25% of patients)**, ***n*** **(%)**
Drooping of one or both eyelids (ptosis)	94 (73.4)
Blurred or double vision (diplopia)	72 (56.2)
Impaired speech (dysarthria)	48 (37.5)
Shortness of breath (dyspnoea)	43 (33.6)
Difficulty chewing	40 (31.2)
Difficulty swallowing (dysphagia)	37 (28.9)
**Most bothersome symptoms**, ***n*** **(%)**	*n* = 115
Drooping of one or both eyelids (ptosis)	94 (81.7)
Blurred or double vision (diplopia)	72 (62.6)
Impaired speech (dysarthria)	48 (41.7)
Shortness of breath (dyspnoea)	17 (14.8)
Difficulty chewing	40 (34.8)
Difficulty swallowing (dysphagia)	37 (32.2)

Of 59/107 patients (55.1%) with physician-reported need for additional support/care, in 78.0% of cases, this was provided by their partner or spouse. Caregiver data are summarized in Supplemental Table 2. Caregivers were mostly partners/spouses [83.3% (*n* = 10/12)].

### 3.3 Humanistic burden

**Figure 1:**
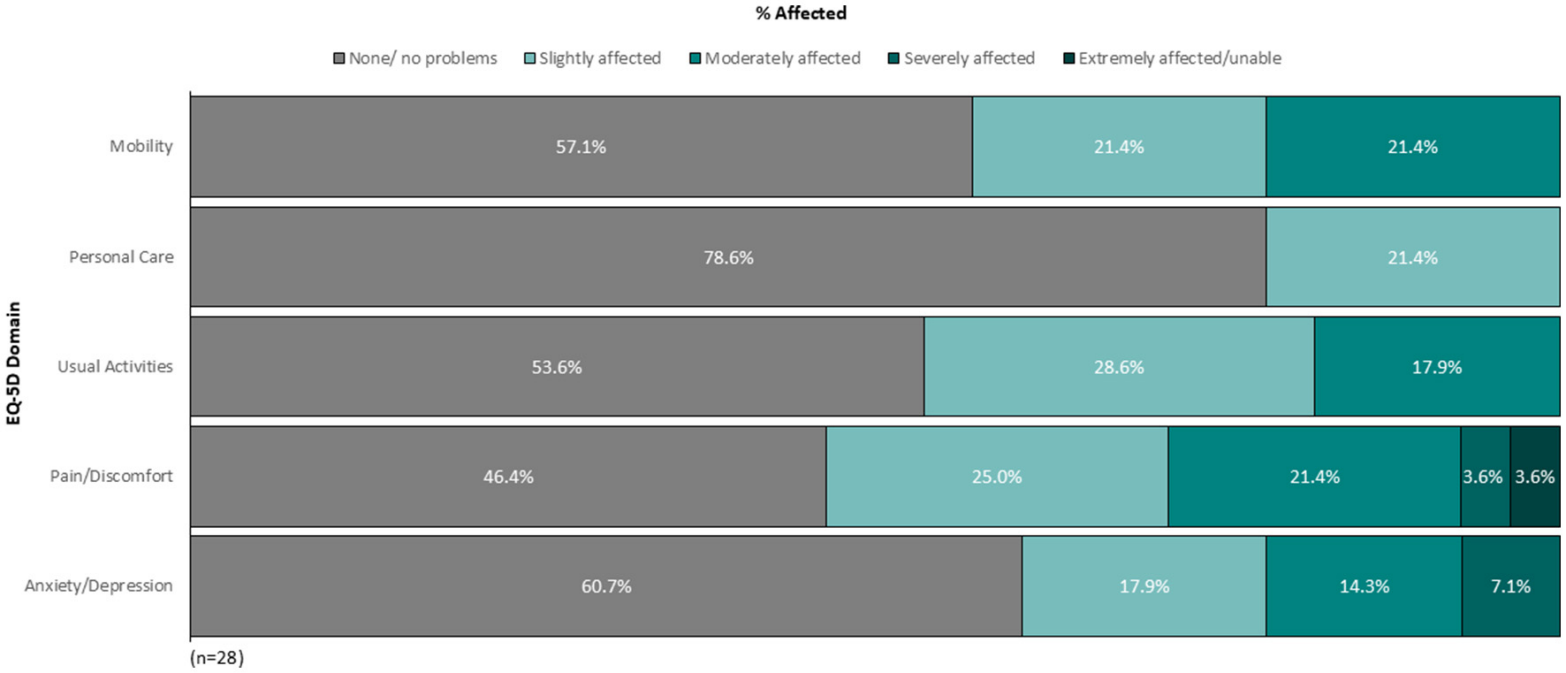
Percentage of patients self-reporting problems on individual EQ-5D-5L domains (*n* = 28).

Patient self-reported HRQoL data (*n* = 29) are shown in Table 3. Patient-reported mean (SD) scores for MG-QOL-15R, EQ-5D-5L utility score, EQ-5D-VAS, and FACIT-Fatigue were 8.5 (7.9), 0.805 (0.190), 66.1 (19.9), and 34.5 (12.4), respectively. On the EQ-5D-5L, patients reported problems with their usual activities [46.4% of patients (*n* = 13/28)], mobility [42.9% (*n* = 12/28)], and self-care [21.4% (*n* = 6/28)], as well as pain/discomfort [53.6% of patients (*n* = 15/28)] and anxiety/depression [39.3% (*n* = 11/28); Figure 1].

**Table 3:** Patient- and caregiver-reported humanistic burden.

**Variable**	**At time of data collection**
**MG-QoL-15R (PSC)**	*n* = 24
Mean (SD)	8.5 (7.9)
**EQ-5D-5L utility score (Japanese tariff; PSC)**	*n* = 28
Mean (SD)	0.805 (0.190)
**EQ-5D-VAS (PSC)**	*n* = 29
Mean (SD)	66.1 (19.9)
**FACIT-Fatigue (PSC)**	*n* = 29
Mean (SD)	34.5 (12.4)
**ZBI-12 (CSC)**	*n* = 12
Mean (SD)	6.9 (5.3)

CSC, caregiver self-completion; FACIT-fatigue, functional assessment of chronic illness therapy—fatigue scale; IQR, interquartile range; MG-ADL, myasthenia gravis activities of daily living scale; MGC, myasthenia gravis composite; MG-QoL-15R, the myasthenia gravis-quality of life 15-revised; PRF, patient record form; PSC, patient self-completion; SD, standard deviation; VAS, visual analog scale; ZBI-12, Zarit Burden interview—short form.

Twelve caregivers completed CSC forms, including the ZBI-12 questionnaire evaluating caregiver burden. The mean (SD) score on the ZBI-12 was 6.9 (5.3; Table 3).

### 3.4 Diagnostic tests and assessments

Supplemental Table 3 presents the tests and assessments that >50% of patients had undergone as part of their diagnosis or initial work-up. The most common tests used at diagnosis were anti-AChR antibody test (92.2% of all patients), neurological examination (89.8%), computed tomography (CT; 86.7%), review of medical history (85.2%), and electromyography (EMG; 83.6%).

Overall, patients underwent a median (IQR) of 15.0 (11.2–17.0) tests/assessments to have their gMG diagnosed/rule out other conditions. For 18.4% of patients (*n* = 21/114), physicians reported that it had taken ≥6 months from symptom onset to a diagnosis of gMG, with nine patients (7.9%) experiencing a delay of ≥1 year. The median (IQR) duration from symptom onset to diagnosis was 2.0 (0.9–4.3) months.

Physicians reported that, prior to diagnosis of gMG, 25.8% of all patients were initially misdiagnosed due to symptoms later attributed to the patient's gMG. The most reported suspected diagnoses or misdiagnoses were amyotrophic lateral sclerosis (5.5% of patients), blepharospasm (4.7%) and chronic inflammatory demyelinating polyneuropathy (3.9%).

### 3.5 Treatment patterns

Almost all patients (98.4%) were receiving maintenance therapy at data collection. These patients had received a median (IQR) of 2.0 (1.0–2.0) lines of treatment. Nonsteroidal immunosuppressant therapies were used at first-line (1L) of therapy in 54.5% of cases (*n* = 67/123). Oral systemic steroids were most used [78.0% of patients at 1L (*n* = 96/123), 77.9% at second-line (*n* = 53/68), and 75.0% at third-line (*n* = 15/20)].

**Figure 2:**
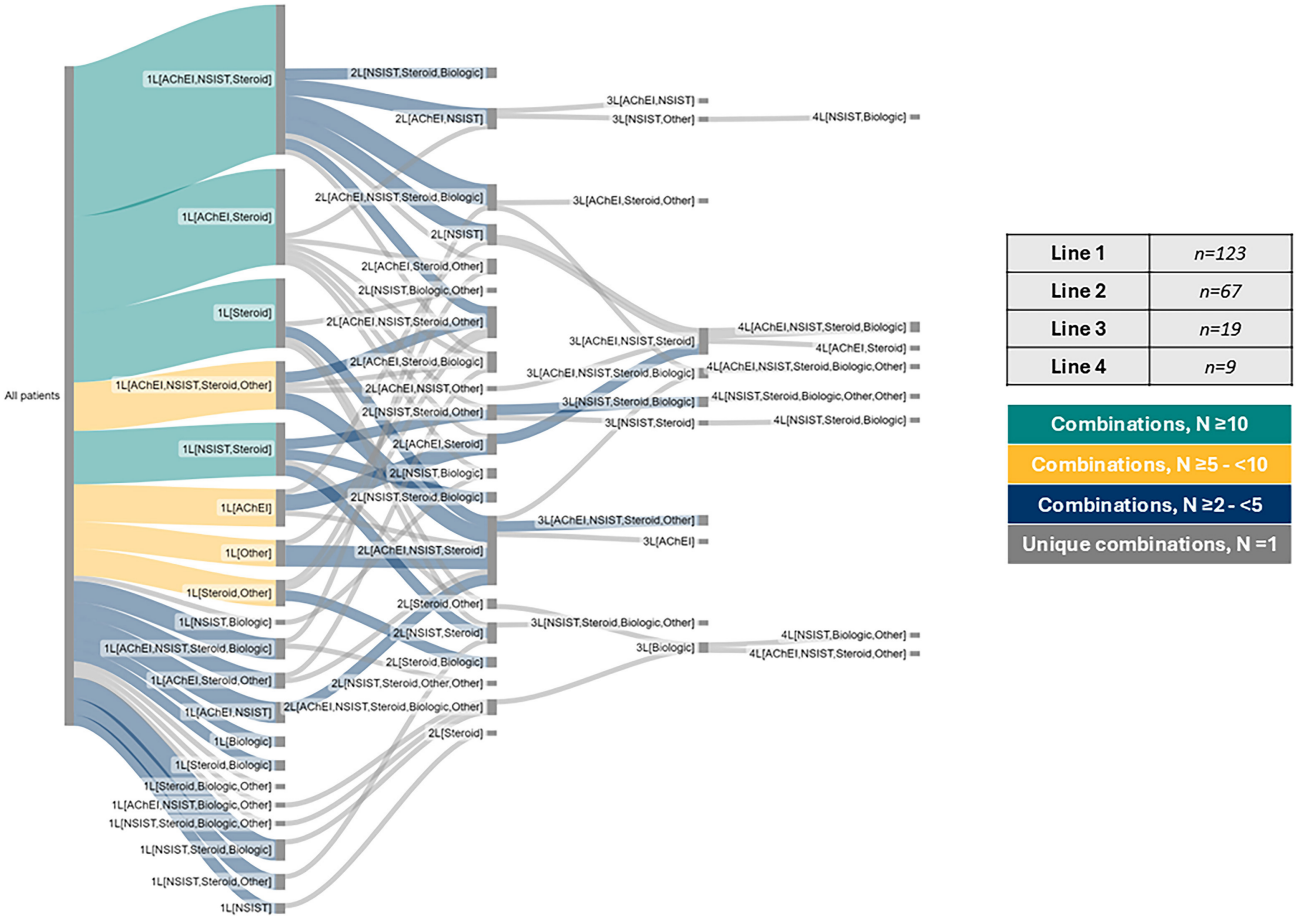
gMG patient treatment patterns to data collection (1L to 4L+). AChEI, Acetylcholinesterase inhibitor; NSIST, Non-steroidal immunosuppressant; IVIG, Intravenous immunoglobulin.

Figure 2 and Table 4 show the detailed treatment patterns in Japan, with Figure 2 summarizing all treatment classes, from 1L to fourth-line (4L) and Table 4 summarizing the top three most frequently prescribed regimen per line of treatment, up to 3L. Combination therapy with acetylcholinesterase inhibitor (AChEi) + nonsteroidal immunosuppressive therapy (NSIST) + steroid was the most prescribed regimen at 1L [22.8% of patients (*n* = 28/123)], 2L [19.1% (*n* = 13/68)], and 3L [25.0% (*n* = 5/20)], respectively.

**Table 4:** Physician-reported top three most frequently prescribed gMG treatment regimens per line of therapy.

**Line of therapy**	**Treatment regimen, *n* (%)**	**At time of data collection**
1L		*n* = 123
	AChEi + NSIST + steroid	28 (22.8)
	AChEi + steroid	18 (14.6)
	Steroid	13 (10.6)
2L		*n* = 68
	AChEi + NSIST + steroid	13 (19.1)
	AChEi + NSIST + steroid + biologic	5 (7.4)
	AChEi + NSIST/NSIST/NSIST + steroid + biologic	4 (5.9)
3L^a^		*n* = 20
	AChEi + NSIST + Steroid	5.0 (25.0)
	Biologic/AChEi + NSIST + Steroid + Biologic/AChEi + NSIST +Steroid + Other/NSIST + Steroid + Biologic	2 (10.0)

^a^No third most frequently prescribed regimen at 3L, all others are n = 1. AChEi, acetylcholinesterase inhibitor; NSIST, nonsteroidal immunosuppressive therapy; 1L, first-line; 2L, second-line; 3L, third-line.

Median (IQR) duration of therapy was 30.2 (5.1–56.2) months for 1L (*n* = 67), 14.0 (5.2–54.0) months (*n* = 51) for 2L, and 6.9 (3.8–63.2) months (*n* = 14) for 3L (Table 5).

**Table 5:** Physician-reported treatment duration and TTNT.

**Duration of any treatment regimen (1L), months**	***n* = 67**
Median (IQR)	30.2 (5.1–56.2)
**TTNT (number of months between the start of 1L to start of 2L)**	***n*** **=** **36**
Median (IQR)	26.6 (0.01–47.3)
**Duration of any treatment regimen (2L), months**	***n*** **=** **51**
Median (IQR)	14.0 (5.2–54.0)
**TTNT (number of months between the start of 2L to start of 3L)**	***n*** **=** **12**
Median (IQR)	9.8 (0.8–28.8)
**Duration of any treatment regimen (3L), months**	***n*** **=** **14**
Median (Q1, Q3)	6.9 (3.8–63.2)
**TTNT (number of months between the start of 3L to start of 4L)**	***n*** **=** **5**
Median (IQR)	17.0 (0.1–67.1)

IQR, interquartile range; TTNT, time to next treatment (defined as duration between the start of 1L to start of 2L, or between the start of 2L to start of 3L, or between the start of 3L to start of 4L); 1L, first-line; 2L, second-line; 3L, third-line.

Table 5 also shows treatment duration and time to next treatment (TTNT) data. TTNT was defined as duration between the start of 1L to start of 2L, or between the start of 2L to start of 3L, or between the start of 3L to start of 4L. TTNT ranged from a median (IQR) of 9.8 [0.8–28.8] months for the start of 2L to start of 3L to 26.6 (0.01–47.3) months for the start of 1L to start of 2L.

At data collection, the top three most prescribed treatments at 1L to 3L was prednisone/prednisolone (prescribed in 77.4, 77.9 and 71.4% of cases at 1L, 2L, and 3L, respectively), tacrolimus (prescribed in 46.8%−64.7% of cases), and pyridostigmine (36.3%−47.6% of cases; Table 6). The median (IQR) dose of prednisone/prednisolone prescribed at data collection was 6.0 (5.0–10.0) mg (*n* = 103). A median (IQR) of 16.8 (6.8–58.2) months had passed since the regimen was initiated.

**Table 6:** Physician-reported top three most frequently prescribed pharmacological therapies as part of any treatment regimen (1L to 3L).

**Top three most frequently prescribed pharmacological therapies as part of any treatment regimen (1L), *n* (%)**	***n* = 124**
Prednisone/prednisolone	96 (77.4)
Tacrolimus	58 (46.8)
Pyridostigmine	46 (37.1)
**Top three most frequently prescribed pharmacological therapies as part of any treatment regimen (2L)**, ***n*** **(%)**	***n*** **=** **68**
Prednisone/prednisolone	53 (77.9)
Tacrolimus	43 (63.2)
Pyridostigmine	26 (38.2)
**Top three most frequently prescribed pharmacological therapies as part of any treatment regimen (3L)**, ***n*** **(%)**	***n*** **=** **21**
Prednisone/prednisolone	15 (71.4)
Tacrolimus	11 (52.4)
Pyridostigmine	10 (47.6)

At data collection, 28.1% of all patients had achieved pharmacological remission. No patients had achieved complete stable remission, and two patients (1.6%) had achieved MM-5 mg.

In 72.2% of cases (*n* = 91/126 patients), physicians reported being somewhat or very satisfied with their patient's maintenance regimen at data collection. The patient-reported equivalent was 48.3% (*n* = 14/29 patients).

#### 3.5.1 Hospitalizations

A total of 32.1% of patients (*n* = 35/109) experienced ≥1 hospitalizations (including hospitalizations for surgery) in relation to their gMG in the 12 months prior to data collection.

Table 7 summarizes the data for the most recent hospitalization for the patients who had a minimum of one hospitalization in the 12 months prior to data collection. The most common reason for the most recent hospitalization was to treat a complication of MG [48.6% of patients (*n* = 17/35)]. All patients (*n* = 31/31) had overnight hospitalizations and spent a median (IQR) of 14.0 (7.0–20.0) nights per admission in hospital.

**Table 7:** Physician-reported most recent hospitalization in the 12 months prior to data collection.

**Reason for the patient's most recent inpatient hospitalization, *n* (%)**	***n* = 35**
To treat a disease complication	17 (48.6)
For surgery	3 (8.6)
To manage a side effect	5 (14.3)
Other reason	10 (28.6)
**Most recent hospitalization type of admission**, ***n*** **(%)**	***n*** **=** **31**
Day	0 (0)
Night	31 (100)
**Number of nights spent in hospital during most recent hospitalization**	***n*** **=** **31**
Median (IQR)	14.0 (7.0–20.0)

IQR, interquartile range; SD, standard deviation.

Most patients [65.0% (*n* = 78/120)] had never experienced a MG-related clinical event. Since diagnosis, 10.8% of patients (*n* = 13/120) had experienced a myasthenic crisis and 17.5% (*n* = 21/120) had experienced an exacerbation of symptoms. Eight patients (6.7%) had experienced both a myasthenic crisis and an exacerbation of symptoms at least once each since diagnosis. Of patients with data on myasthenic crises (*n* = 16), three (18.8%) had experienced a myasthenic crisis in the 12 months prior to data collection. Of 26 patients with data on exacerbation of symptoms in the 12 months prior to data collection, 13 patients had not experienced any exacerbation during this time, whilst eight (30.8%), four (15.4%), and one (3.8%) had experienced 1, 2, or 3+ exacerbations, respectively.

## 4 Discussion

In this study, 98% of patients were on maintenance treatment, however, we observed impaired HRQoL, such as difficulties with usual activities and mobility.

Consistent with previous reports (6), the present real-world survey revealed impaired HRQoL in patients with gMG prescribed maintenance therapy. Patients reported difficulties with their usual activities (46% of patients), mobility (43%), and self-care (21%), as well as pain/discomfort (54% of patients) and anxiety/depression (39%) on the EQ-5D-5L questionnaire.

Furthermore, the adjusted mean EQ-5D-5L value (Japanese tariff) in the present gMG population was 0.805, which is below the Japanese general population norms, which ranged from 0.821 (male) and 0.774 (female) in the group aged 80–89 years to 0.978 (male) and 0.967 (female) in the group aged 16–19 years (29). The mean EQ-5D-VAS score was 66.1 in the present study whereas it was 84.3 (male) and 83.6 (female) in the youngest age group, and 70.3 (male) and 68.1 (female) in the oldest age group in the Japanese general population (29). These findings indicate that despite maintenance treatment in 98% of patients in this study cohort, unmet needs remained in gMG patients' daily lives, with HRQoL still substantially impacted. In a previous survey in Japan, MG patients commonly identified exercise (74%), work (68%), hobbies (60%), travel (60%), and socializing with friends (60%) as being difficult to perform (6).

We also observed mild caregiver burden in the current study, as assessed by the ZBI-12 (a total score of 7/48). However, no firm conclusions can be drawn, due to the small number of caregivers (*n* = 12) included in the analysis. Further investigation in this area is warranted.

The most recent Japanese treatment guidelines (1) recommend EFT (plasmapheresis, IVIg, IVMP, or a combination of these) for earlier achievement of MM-5 mg, which is the main goal of MG treatment. In addition to preventing exacerbation of symptoms, FT should be used to reduce the use of oral steroids or as maintenance therapy (1, 12, 30), as long-term therapy with high-dose steroids is associated with various adverse reactions or side effects and negative impact on HRQoL (1, 30).

Although almost one-third of patients had achieved pharmacological remission, only two patients had achieved MM-5 mg and no patients were in complete stable remission at data collection; however, the latter is rarely achieved (1, 31). Evidence from JAMG-R indicates that implementing EFT together with early use of calcineurin inhibitors increases the likelihood of achieving the treatment target of MM-5 mg in patients with gMG (32); however, our real-world findings suggest that achieving MM-5 mg with existing therapies remains challenging, a finding also recently echoed by Teranishi et al. (9).

The present study revealed a complex treatment landscape in this cohort from Japan, with evidence that nonsteroidal immunosuppressant therapies are being used from 1L (in line with the most recent Japanese clinical guidelines) (1). The most used drugs in the present study were oral steroids, with prednisone/prednisolone being prescribed in over three-quarters of patients regardless of line of treatment (1L, 2L, or 3L). It was also apparent that steroids were mostly used in combination with other treatments. Similarly, Kawaguchi et al. (6) previously reported the use of steroids in 72% of MG patients surveyed in Japan, followed by immunosuppressants (60%), and AChEi (54%), while Teranishi et al. (9) found that most gMG patients in their Japanese database study were treated with combinations of AChEi, oral steroids, and/or NSISTs. Interestingly, in the present study, compared with physicians (72%), less than half of patients reported being somewhat or very satisfied with their maintenance treatment regimen at data collection, which is similar to previous findings in the US and Europe, with physicians reporting being very satisfied with their patient's current maintenance treatment more frequently (64%) than their patients (39%) (33). Reasons for possible discordance between patient and physician opinion were not explored in the current study but this would be an interesting topic for future study.

Patient demographics and clinical characteristics in our study were largely similar to those reported by the Japanese myasthenia gravis registry (JAMG-R) (12), in which mean patient age was 60 years, 60% of patients were female, 44% were MGFA Class II, 82% were anti-AChR, and 3% were anti-MuSK positive. However, the proportion of seronegative patients in our study was 4% compared with 11% reported by Suzuki et al. in their 2021 survey of the JAMG-R. This difference may be due to better diagnostic testing, in line with an increase in the availability of seropositive-specific treatments.

The present study has several limitations. Data may not reflect the general gMG population, as participating patients had to have visited their physician to be included, therefore, patients who visited their physician more frequently and were more severely affected would have been more likely to be included in the DSP. The DSP is not based on a true random sample of physicians or patients—while minimal inclusion criteria governed the selection of the participating physicians, participation was influenced by willingness to complete the survey, and practical considerations of geographical location. Physicians were asked to provide data for a consecutive series of patients to avoid selection bias, but no formal patient selection verification procedures were conducted. Recall bias, a common limitation of surveys, might also have affected participants' responses to the questionnaires. However, physicians did have the ability to refer to patients' medical charts to minimize this.

It should also be noted that the small number of patients and caregivers who completed the PSC or CSC form limits the generalizability of the findings from the patient- and caregiver-reported data. Lastly, although the MGFA classification is the most widely recognized classification system for MG clinical state, this evaluation may have varied by physician, as the classification system is not used to assess current disease state but worst disease state (i.e., maximum disease severity).

Notwithstanding these limitations, to our knowledge this is the first study to use both patient- and physician-reported data to systematically describe the disease burden, quality of life, and treatment patterns of a cohort of Japanese patients with gMG in real-world settings, laying a foundation for future research and providing a reference for clinicians to optimize treatment strategies.

In summary, this study revealed remaining unmet needs with regards to physical symptoms and HRQoL in this cohort of Japanese patients with gMG. Despite most patients being on maintenance therapy, including novel treatments, the present data indicated impaired usual activities and mobility. Novel gMG treatments with potentially stable effectiveness (34) may help mitigate some of these remaining unmet needs, while providing a steroid-sparing effect.

## Data Availability

The datasets presented in this article are not readily available because all data, i.e., methodology, materials, data and data analysis, that support the findings of this survey are the intellectual property of Adelphi Real World. Requests to access the datasets should be directed to Joe Conyers, joe.conyers@adelphigroup.com.
