# Supplementary material for: Humanistic burden and medical care patterns of real-world patients with myasthenia gravis in Japan
Source: Front Neurol. 2025 Dec 11;16:1673297. doi: 10.3389/fneur.2025.1673297 (PMC12742322; doi:10.3389/fneur.2025.1673297)
Supplement: Supplementary file 1 [file Table_1.docx]

# Supplemental Table 1. MG-ADL and MGC total scores by MGFA Class

|  | **MGFA**  **Class I** | **MGFA**  **Class II** | **MGFA Class**  **III** | **MGFA Class**  **IV** | **MGFA Class V** |
| --- | --- | --- | --- | --- | --- |
|  | **n=36** | **n=76** | **n=10** | **n=5** | **n=1** |
| **MG-ADL total score** |  |  |  |  |  |
| Mean (SD) | 1.5 (1.8) | 4.2 (3.0) | 7.6 (2.7) | 15.0 (3.0) | 18.0 (0.0) |
| **MCG total score** |  |  |  |  |  |
| Mean (SD) | 1.2 (2.1) | 6.1 (5.4) | 16.0 (6.0) | 33.8 (5.2) | 30.0 (0.0) |

MG-ADL; Myasthenia Gravis Activities of Daily Living Scale; MGC: Myasthenia Gravis Composite; MGFA: Myasthenia Gravis Foundation of America; SD: standard deviation

# Supplemental Table 2a. Physician-reported caregiver data

| **Patient has caregiver/requires additional support, n (%)** | **n=107** |
| --- | --- |
| Yes | 59 (55.1) |
| No | 48 (44.9) |
| Don’t know | 21 (19.6) |
| **Caregiver relationship to patient, n (%)** | **n=59** |
| Partner/spouse | 46 (78.0) |
| Son/daughter ≥18 years | 4 (6.8) |
| Parent/guardian | 7 (11.9) |
| Other relative(s) | 1 (1.7) |
| Other non-professional caregiver(s) | 1 (1.7) |
| Professional caregiver(s) | 8 (13.6) |

# Supplemental Table 2b. Caregiver-reported caregiver demographics

|  | **At time of data collection**  **n=12** |
| --- | --- |
| **Age (in years)** |  |
| Mean (SD) | 57.5 (14.3) |
| **Sex, n (%)** |  |
| Male | 7 (58.3) |
| Female | 5 (41.7) |
| **Relationship with patient, n (%)** |  |
| Partner/spouse | 10 (83.3) |
| Parent | 1 (8.3) |
| Professional/paid caregiver | 1 (8.3) |

SD: standard deviation

# Supplemental Table 3. Physician-reported tests and assessments conducted as part of the diagnosis process

| **Tests/assessments conducted in diagnosis or initial work-up (reported for >50% of patients), n (%)** | **n=128** |
| --- | --- |
| Anti-AChR antibody test | 118 (92.2) |
| Neurological examination | 115 (89.8) |
| CT scan | 111 (86.7) |
| Review of medical history | 109 (85.2) |
| Repetitive nerve stimulation tests (EMG) | 107 (83.6) |
| Blood pressure | 99 (77.3) |
| MG-ADL scale | 97 (75.8) |
| Liver function testing | 96 (75.0) |
| Full blood count | 96 (75.0) |
| CRP test | 94 (73.4) |
| Thyroid function testing | 90 (70.3) |
| Pulmonary function tests | 84 (65.6) |
| Physical examination | 84 (65.6) |
| QMG scale | 74 (57.8) |
| Edrophonium test/Tensilon test | 70 (54.7) |
| Anti-MuSK antibody test | 69 (53.9) |
| MRI scan | 69 (53.9) |

AChR: acetylcholine receptor; CRP: C-reactive protein; CT: computed tomography; EMG: electromyography; MG-ADL: MG Activities of Daily Living; MRI: magnetic resonance imaging; QMG: Quantitative Myasthenia Gravis; MuSK: muscle-speciﬁc kinase
